# Urothelial carcinoma arising within a congenital bladder diverticulum in an adult male: A rare case report and literature review

**DOI:** 10.1016/j.amsu.2022.103666

**Published:** 2022-04-22

**Authors:** Seif-Aldin Abdulrahman, Ibrahim Muhammad, Ali Abdulrahman, Khidr Raslan, Zuheir Alshehabi

**Affiliations:** a Faculty of Medicine, Cancer Research Center, Tishreen University, Latakia, Syria; b Department of Urology, Tishreen University Hospital, Latakia, Syria; c Department of Pathology, Cancer Research Center, Tishreen University, Latakia, Syria

**Keywords:** Urothelial carcinoma, Intradiverticular tumor, Congenital bladder diverticulum, Bladder cancer, Case report

## Abstract

**Introduction and importance:**

Neoplasms arising from vesical diverticula are rare clinical entities known as intradiverticular bladder tumors. The bladder diverticulum harboring these tumors can be congenital or acquired. Congenital diverticula are predominantly found in children and are extremely rare in adults.

**Case presentation:**

A 56-year-old male admitted to our hospital with a 10-days history of painless gross hematuria and blood clots. Physical examination and vital signs were unremarkable. Radiologic examination revealed a bladder diverticulum with a mass inside it. Pathological examination confirmed the diagnosis of TCC tumor arising from congenital bladder diverticula.

**Clinical discussion:**

While intradiverticular bladder tumors account for only 1% of all bladder tumors، and the finding of a congenital bladder diverticulum is a rare anomaly in adults, the concurrence of a tumor arising within a congenital bladder diverticulum is almost non-existent in adults.

**Conclusion:**

We aimed to present a rare case of urothelial carcinoma arising from a congenital diverticulum in an adult while highlighting the diagnostic and therapeutic options used in managing such tumors.

## Highlights

• Congenital diverticula are predominantly found in children and are extremely rare in adults.
• The concurrence of a tumor arising within a congenital bladder diverticulum is almost non-existent in adults.
• Physicians should always consider the possibility of tumors developing within these diverticula.

## 1 Introduction

Bladder diverticula are herniations of bladder mucosa through congenital or acquired weakness in the musculature of the bladder wall [1].

This results in a thin-walled sack with a narrow neck, leading to incomplete emptying during micturition along with urinary stasis [2].

Bladder diverticula are either congenital or acquired. Acquired diverticula are more common and typically seen in adults, predominantly males over 50 years of age [3,4]. On the other hand, congenital bladder diverticulum is a development failure of the detrusor typically resulting from its hypoplasia [5]. The prevalence of congenital diverticula is reported to be around 1.7% with a peak incidence among children under the age of 10 years [1,3].

From a histological point of view, congenital diverticula encompass all layers of bladder wall whereas acquired diverticula are devoid of muscularis propria [6,7].

Given the association between bladder diverticula and urinary stasis, there is an increased risk of urolithiasis, chronic inflammation, and recurrent infections [8]. Urinary stasis also prolongs the bladder mucosa's contact with potential carcinogens. This potentially explains the increased risk of neoplasms within the bladder diverticula compared to the main bladder [9].

The incidence of primary neoplasms arising from bladder diverticula ranges from 0.8% to 13% [10].

However, due to most bladder diverticula being asymptomatic, the true prevalence remains unknown.

Intradiverticular bladder tumors represent 1% of all bladder tumors and are rarely seen in general practice. Nevertheless, they are more common in males than females with a ratio of (9:1) [11].

Herein, we present the first case from Syria-to our knowledge-of an intradiverticular tumor arising from a congenital bladder diverticulum in a 56-year-old male. This case was reported according to the SCARE 2020 criteria [12].

## 2 Case Presentation

A 56-year-old male admitted to the Urology Department of our hospital with a 10-days history of painless gross hematuria and blood clots.

Physical examination and vital signs were unremarkable. The symptoms were not associated with dysuria, fever or weight loss.

Medical and Family History were unremarkable. Clinically, bladder cancer and lower urinary tract infection were considered as differential diagnoses. The blood test results were all within normal limits.

Urine analysis was positive for red blood cells whereas urine cytology for cancer cells was negative.

**Fig. 1:**
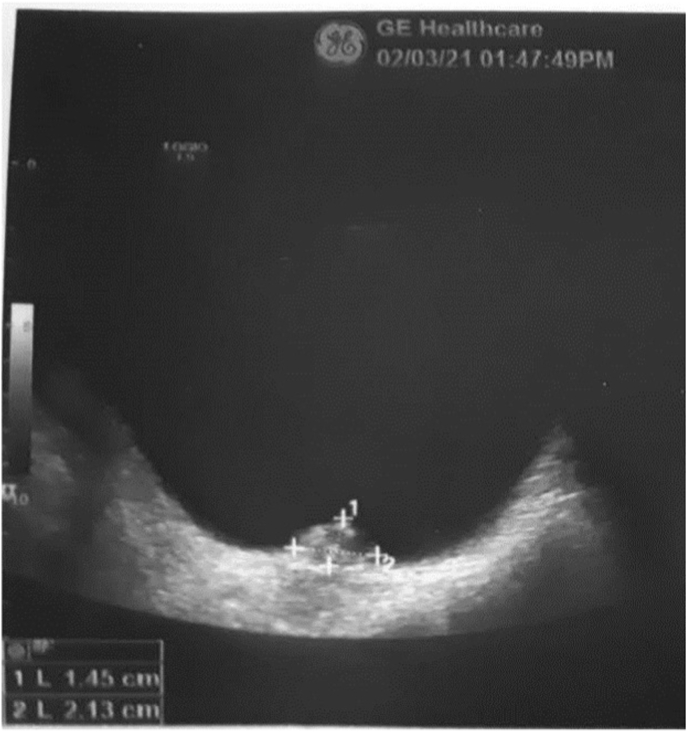
Ultrasound of the bladder showing a hyperechoic mass (14.5 mm × 21.3 mm) with multiple echogenic foci.

Ultrasound examination of the abdomen and pelvis revealed a pedunculated mass measuring (21 × 14 mm) on the right posterior wall of the bladder near the ureterovesical junction ***(***Fig. 1).

**Fig. 2:**
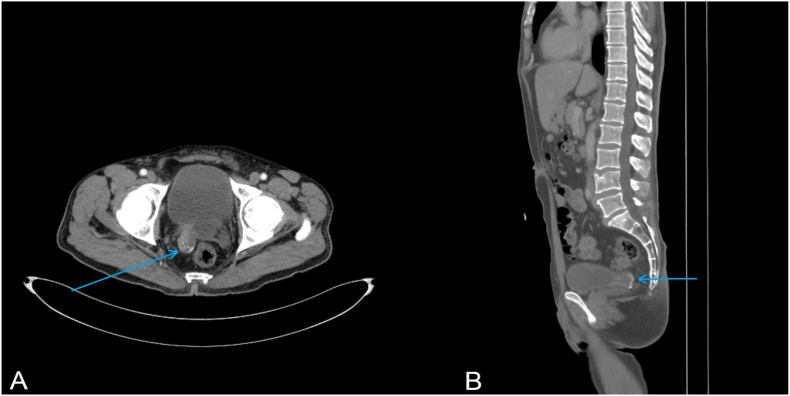
**A** and **B** CT scan revealing a small bladder diverticulum (26mm × 37mm) on the right posterior wall of the bladder.

Later on, a CT scan was carried out and revealed a bladder diverticulum (26 × 37 mm) with a mass inside it reaching out to the adjacent bladder wall and the right ureterovesical junction. The CT scan also revealed thickening of the bladder dome (7 mm) in addition to 3 enlarged lymph nodes measuring up to (18 mm) in the retroperitoneal space, left to the abdominal aorta ***(***Fig. 2).

Cystoscopy did not reveal the diverticulum due to poor visibility in the bladder.

Based on these findings, the medical team recommended surgical intervention and the surgeon (Dr.Raslan) performed radical cystectomy with pelvic lymphadenectomy.

**Fig. 3:**
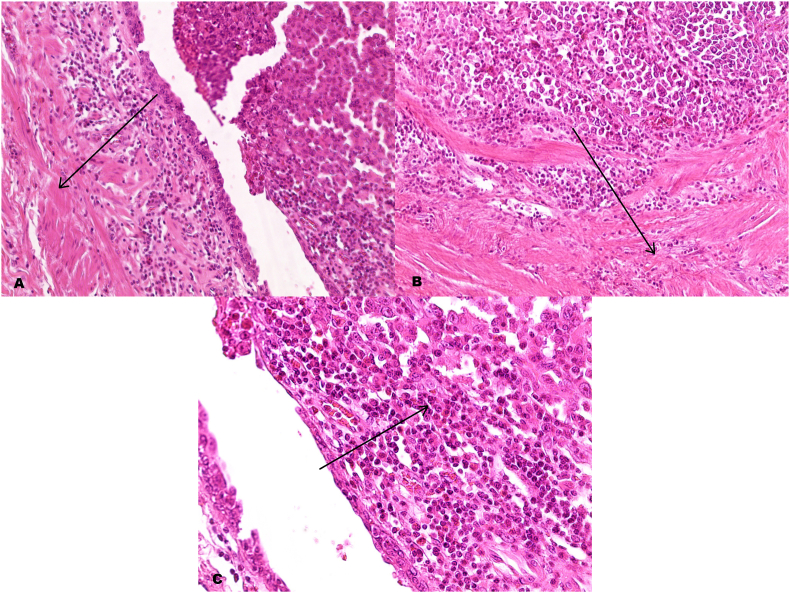
A and ***B*** revealing the tumor cells invading the muscle coat of the diverticulum. (H&E stain **A** original magnification ×100 **B** original magnification x200). ***C*** revealing non-papillary proliferation of urothelial cells with hyperchromatic nuclei and occasional bizarre mitotic figures (H&E original magnification ×400).

The pathological examination of the diverticulum demonstrated its congenital origin by revealing the muscular layer. Biopsies taken from the tumor mass inside the diverticulum revealed high-grade transitional cell carcinoma invading the perivesical fat (T3a). Other biopsies taken from the main bladder mass also showed high-grade transitional cell carcinoma, infiltrating the subepithelial connective tissue (T1) with the invasion of one regional lymph node (N1). Margins of surgical resection including the prostate and the left ureter were free from neoplastic cells ***(***Fig. 3***).***

Surgery was followed up with systemic adjuvant chemotherapy which included Gemcitabine 1100 mg/m^2^ over 30 minutes intravenously on days 1 and 8, followed on day 1 by Carboplatin 320 mg/m^2^ intravenously over 1 h. Treatment cycles were repeated every 21 days for five cycles.

The patient was put on a regular follow-up with CT scan every 3 months, however, he did not return to our hospital.

## 3 Discussion

Intradiverticular bladder tumors are rare entities that were first described by Targett in 1896 and only represent 1% of all bladder tumors [13].

They predominantly occur in acquired diverticula which present as multiple lesions and are generally found in adults.

In comparison, congenital diverticula –as our case-are mainly solitary, typically found near the uertero-vesical junction and rarely present in adults [[14], [15]].

While the presence of congenital diverticula in adults and intradiverticular bladder tumors are both rare clinical entities, the concurrence of carcinoma arising from a congenital diverticulum in adults is almost non-existent [9].

Histologically, urothelial carcinoma is the most common malignancy in the bladder diverticulum (78%), followed by squamous cell carcinoma (17%), mixed urothelial and squamous cell carcinoma (2%) and adenocarcinoma (2%). On the other hand, regardless of the histological subtype, an intradiverticular bladder tumor poses a greater diagnostic challenge given that the majority of diverticula are asymptomatic, as well as a greater management challenge because it tends to be of a higher grade compared to the main bladder tumor [9,16].

The most common symptom in patients with intradiverticular bladder tumors is painless hematuria [[17], [18]]. However, a negative sample offers little value in excluding the diagnosis because the hematuria can be intermittent. Other common symptoms include urinary tract infection and urinary retention [[19], [20], [21]].

The procedures used to diagnose intradiverticular bladder tumors are practically identical to those used for the diagnosis of main bladder cancer and include urine cytology, cystoscopy, imaging and pathological examination [9]. Urine cytology is the examination of a urine sample to detect exfoliated cancer cells. Although this test is non-invasive and highly specific, its sensitivity is poor in low-grade tumors and the interpretation of the specimen depends on the examiner's experience [9,[21], [22]].

Cystoscopy allows for direct visualization of the bladder cavity and is considered an acceptable initial examination in detecting bladder tumors and bladder diverticula. Nevertheless, cystoscopy often fails to identify the tumor mass in the diverticulum, particularly when obstructed by a tight orifice, making the diverticulum difficult to access [[23], [24], [25]].

In general, the first imaging study performed on patients with hematuria is ultrasound (US) [24].

Currently, there is no data available for the value of ultrasound and magnetic resonance imaging (MRI) [24]. In contrast, computed tomography (CT) offers great value in establishing the diagnosis along with the tumor stage [22,24].

Finally, the conclusive diagnosis of malignancy is established pathologically through a biopsy of the lesion [9,18].

After confirming the diagnosis, several treatment options can be considered including transurethral resection, partial cystectomy, radical cystectomy and multimodal therapy. Transurethral resection (TUR) of the bladder is a procedure used for diagnosing bladder tumors and removing the tumor tissue [9].

TUR is reserved for non-invasive tumors (stage Ta) and is accompanied with an increased risk of bladder perforation [24]. When complete TUR is not possible in low-grade tumors due to high risk of perforation, partial cystectomy or diverticulectomy is recommended. It is also preferred when endoscopy is met with a tight diverticular orifice, obstructing thorough endoscopic resection. Compared to TUR, partial cystectomy or diverticulectomy allows for better disease management while avoiding the risk of perforation. In contrast, more advanced cases require radical cystectomy which is associated with a significant mortality rate along with the need for urinary diversion, impacting the patient's quality of life [9].

Multimodal therapy is the use of at least two of the following modalities: chemotherapy, radiation therapy and diverticulectomy [9,24].

Garzotto et al. applied this treatment in their study to a group of 9 patients and observed an 89% 4-year disease-free period [16,25].

This approach demonstrated superior efficacy and showed promising results. Walker et al. recommended in their review the integration of adjuvant or neoadjuvant systemic therapy along with surgery in high-risk patients [9].

An important note to keep in mind when determining the treatment plan is the risk of clinical understaging. The aforementioned risk was observed in 55% of Ta and T1 patients undergoing radical cystectomy [24].

## 4 Conclusion

Congenital bladder diverticula are rarely found in adults and present a significant diagnostic challenge due to mostly being asymptomatic.

Physicians should always consider the possibility of tumors developing within these diverticula.

In this case, we reviewed the diagnostic and therapeutic options and recommendations while highlighting the lack of available data on the optimal management of intradiverticular bladder tumors.

## Ethical approval

Not applicable. It's a case report.

## Sources of funding

None.

## Contributions

SA and IM: Drafted the manuscript and collected the patient's data.

AA: Collected the data and participated in drafting the manuscript.

KR: Performed the surgical operation and provided the patient's data.

ZA: The mentor and guarantor, performed the pathological examination, critically revised the article and approved the final manuscript.

SA and IM contributed equally to this work.

## Registration of research studies

Not applicable. It's a case report.

## Guarantor

Dr.Zuheir Alshehabi.

## Consent

Written informed consent was obtained from the patient for publication of this case report and accompanying images. A copy of the written consent is available for review by the Editor-in-Chief of this journal on request.

## Availability of data and materials

Data and material are available on reasonable request from the guarantor and mentor of this study Prof. Alshehabi.

## Provenance and peer review

Not commissioned, externally peer-reviewed.

## Declaration of competing interest

None.
